# Preparation and Use of Turn-on Fluorescent Probe for Detection and Live Cell Imaging of Vitamin D Receptor as a Target Protein

**DOI:** 10.1016/j.xpro.2020.100036

**Published:** 2020-06-03

**Authors:** Hiroyuki Kojima, Toshimasa Itoh

**Affiliations:** 1 Showa Pharmaceutical University, 3-3165 Higashi-Tamagawagakuen, Machida, Tokyo 194-8543, Japan

## Abstract

Turn-on fluorescent probe mediated by conjugate addition and cyclization (TCC probe) is a small molecule that reacts with a protein of interest in cells. TCC probe is applicable to various types of proteins by exchanging the ligand unit for target proteins. TCC probes are a potent tool for molecular imaging and chemical proteomics. This protocol describes the synthesis of a TCC probe via unstable intermediate and how to use this probe to visualize vitamin D receptor as a target protein.

For complete details on the use and execution of this protocol, please refer to Kojima et al. (2020).

## Highlights

• Protocol to prepare a turn-on fluorescent probe for proteins
• Method for live cell imaging of a protein of interest
• Protocol can be used for chemical proteomics

## Graphical Abstract

**Figure:**
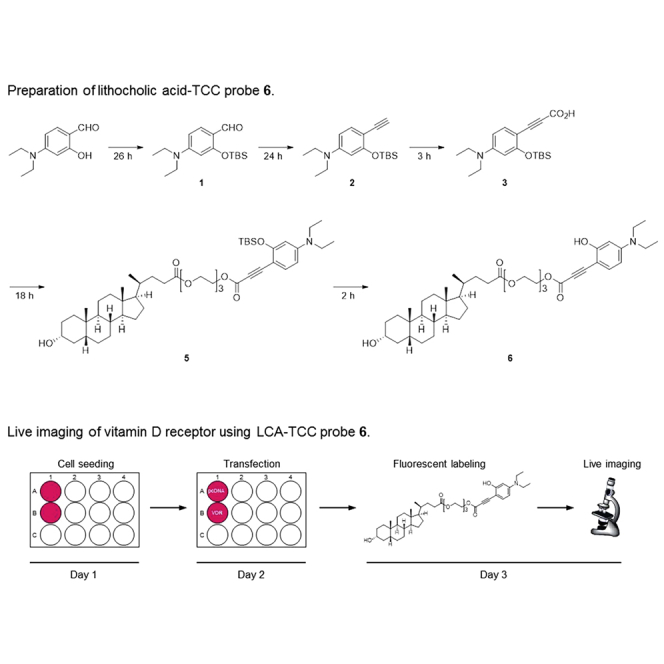

## BEFORE YOU BEGIN

***Note:*** All reagents were purchased from commercial suppliers and used without further purification.

**CRITICAL:** Perform all chemical reactions in an oven-dried glassware under inert gas atmosphere.

**CRITICAL:** Use anhydrous organic solvents for all chemical reactions.

### Reaction Flask

**Timing: 1 h**

1. Oven-dry the reaction flask and cool it down to room temperature (15°C–25°C) while it is in vacuum desiccator.

### Syringes

**Timing: 1 h**

2. Clean syringes and dry out in vacuum desiccator.

### Cell Culture Medium

**Timing: 30 min**

3. Mix 500 mL of DMEM cell culture medium, 50 mL of fetal bovine serum, 5 mL of penicillin-streptomycin mixed solution (penicillin: 10,000 units/mL, streptomycin 10,000 μg/mL) in a sterile environment.

### Poly-*L*-lysine Coated Cover Slips

**Timing: 24 h**

4. Place cover slips into a new sterile 24-well cell culture plate.
5. Add 0.5 mL of 70% EtOH to each well to sterilize cover slips. Incubate the plate at room temperature (15°C–25°C) for 10 min.
6. Remove the 70% EtOH and leave it open in a sterile environment for complete drying.
7. Prepare the stock solution by dissolving poly-*L*-lysine hydrobromide powder in distilled water at a 1 mg/mL concentration. Divide the solution into aliquots and store at -20°C. Prepare the working solution freshly before coating the culture surfaces by diluting the stock 1:100 in distilled water.
8. Coat each cover slip by incubating it with 0.5 mL of poly-*L*-lysine working solution for 12 h in an incubator with 5% CO_2_ at 37°C.
9. Rinse each well five times with 0.5 mL of distilled water and leave it open in a sterile environment for complete drying.

## KEY RESOURCES TABLE

**Table:** 

REAGENT or RESOURCE	SOURCE	IDENTIFIER
**Chemicals, Peptides, and Recombinant Proteins**		

4-(Diethylamino)salicylaldehyde	Tokyo Chemical Industry	Cat#D1752
*tert*-Butyldimethylchlorosilane	Tokyo Chemical Industry	Cat#B0995
Imidazole	Tokyo Chemical Industry	Cat#I0288
Diisopropylamine	Tokyo Chemical Industry	Cat#D0925
*n*-Butyllithium in cyclohexane (2.0 mol/L)	Sigma-Aldrich	Cat#302120-4X25ML
(Trimethylsilyl)diazomethane in diethyl ether (2.0 mol/L)	Sigma-Aldrich	Cat#527254
Lithocholic acid	Tokyo Chemical Industry	Cat#L0089
4-Dimethylaminopyridine	Tokyo Chemical Industry	Cat#D1450
*N,N*′-Dicyclohexylcarbodiimide	Nacalai Tesque	Cat#11914-42
Hydrogen chloride in ethyl acetate (1.0 mol/L)	Tokyo Chemical Industry	Cat#H1060
Tetrabutylammonium fluoride in tetrahydrofuran (1.0 mol/L)	Tokyo Chemical Industry	Cat#T1338
Acetone	Kokusan Chemical	Cat#2140039
Chloroform	Kanto Chemical	Cat#07278-80
Chloroform-D	FUJIFILM Wako Pure Chemical	Cat#559-17811
Dichloromethane, dehydrated	Kanto Chemical	Cat#11338-84
*N,N*-Dimethylformamide, super dehydrated	FUJIFILM Wako Pure Chemical	Cat#045-32365
Ethanol	Nacalai Tesque	Cat#14713-53
Ethyl acetate	Nacalai Tesque	Cat#14622-14
Hexane	Nacalai Tesque	Cat#17921-04
Tetrahydrofuran, dehydrated	Kanto Chemical	Cat#40993-85
Magnesium sulfate (anhydrous)	FUJIFILM Wako Pure Chemical	Cat#137-12335
Sodium sulfate (anhydrous)	FUJIFILM Wako Pure Chemical	Cat#194-03355
Silica gel60(sphere:40~50 μm)NH_2_	Kanto Chemical	Cat#37567-79
Silica gel60N(sphere:40~50 μm)	Kanto Chemical	Cat#37563-85
Silica gel 70 F254 TLC Plate-Wako (0.25 mm thickness)	FUJIFILM Wako Pure Chemical	Cat#193-17811
Argon gas	Koike Sanso Kogyo	N/A
CO_2_ gas	Koike Sanso Kogyo	N/A
Compound **1**	Kojima et al., 2020	N/A
Compound **2**	Kojima et al., 2020	N/A
Compound **3**	Kojima et al., 2020	N/A
Compound **4**	Kojima et al., 2020	N/A
Compound **5**	Kojima et al., 2020	N/A
Compound **6**	Kojima et al., 2020	N/A
Dulbecco’s Modified Eagle’s Medium – high glucose	Sigma-Aldrich	Cat#D5796-500ML
DMEM/Ham’s F-12	Nacalai Tesque	Cat#05177-15
Fetal bovine serum	Sigma-Aldrich	Cat#F7524
Penicillin-Streptomycin Mixed Solution	Nacalai Tesque	Cat#09367-34
PBS	Nacalai Tesque	Cat#07269-84
Trypsin-EDTA	Nacalai Tesque	Cat#32777-44
Opti-MEM	Gibco	Cat#31985070
TransIT-LT1	Mirus	Cat#MIR2300
Poly-*L*-lysine hydrobromide	Sigma-Aldrich	Cat#P9155
1α,25-Dihydroxyvitamin D_3_	Tokyo Chemical Industry	Cat#C3078

**Experimental Models: Cell Lines**		

HeLa	RIKEN BRC	N/A

**Recombinant DNA**		

pCMV3-N-OFPSpark-VDR	Sino Biological	Cat#HG12025-ANR
pcDNA3.1	Kojima et al., 2020	N/A

**Other**		

50 mL Round-bottom flask	ISHI Shouten	N/A
10 mL Two-neck round-bottom flask	ISHI Shouten	N/A
25 mL Two-neck round-bottom flask	ISHI Shouten	N/A
50 mL Two-neck round-bottom flask	ISHI Shouten	N/A
100 mL Two-neck round-bottom flask	ISHI Shouten	N/A
Microsyringe 250 μL	Hamilton	Cat#4015-11025
Gas tight syringe 1 mL	Hamilton	Cat#4015-54001
Gas tight syringe 5 mL	Hamilton	Cat#4015-54005
Gas tight syringe 10 mL	Hamilton	Cat#4015-54010
Magnetic stirring bar	N/A	N/A
Magnetic Stirrer	ISHII Shouten	N/A
Sleeve stopper septa	Sigma-Aldrich	Cat#Z565717-100EA
Balloon	DAISO	Cat#4902510010387
Vacuum desiccator	N/A	N/A
Cannula	N/A	N/A
Separatory funnel	ISHI Shouten	N/A
Column	ISHI Shouten	N/A
Rotary evaporator	EYELA	Cat#N-1200A
Bruker AVANCE 300 NMR	Bruker	N/A
AccuTOF LC-plus JMS-T100JP (ESI)	JEOL	N/A
10-cm cell culture dish	IWAKI	Cat#3020-100
12-Well cell culture plate	Greiner Bio-One	Cat#665180
24-Well cell culture plate	Greiner Bio-One	Cat#662-160
Round cover glass	Matsunami Glass	Cat#C012001
Glass bottom dish	MatTek	Cat#P35G-1.5-14-C
Hemocytometer	NanoEntek	Cat#DHC-N01
CO_2_ incubator	SANYO	Cat#MCO-174IC
Nikon ECLIPSE Ti confocal microscope	Nikon	N/A

## MATERIALS AND EQUIPMENT

• Nikon ECLIPSE Ti confocal microscope with a 60× water-immersion objective for fluorescence live-cell imaging.
• For LCA-TCC probe imaging, excitation wavelength: 403.2 nm; emission wavelength: between 425 and 475 nm.
• For VDR-OFP imaging, excitation wavelength: 561.6 nm; emission wavelength: between 570 and 620 nm.
• Acquire all images in a 1024 × 1024-pixel format.

## STEP-BY-STEP METHOD DETAILS

### Synthesis of 2-((*tert*-Butyldimethylsilyl)oxy)-4-(diethylamino)benzaldehyde (**1**)

**Timing: 26 h**

**Scheme 1:**
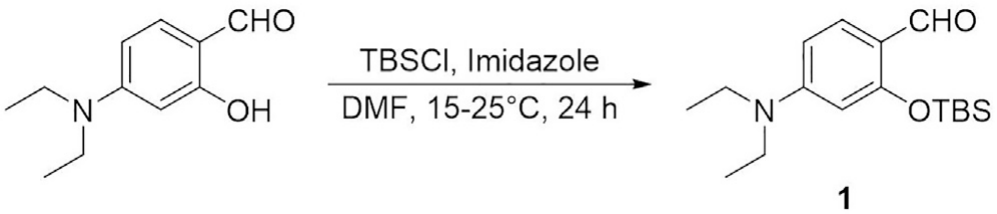
Synthesis of Compound **1**

This step describes how to prepare the compound **1** (Wang et al., 2014). See Scheme 1.

1. Weigh 1.01 g of 4-(diethylamino)salicylaldehyde (5.21 mmol) in a 50 mL round-bottom flask containing a magnetic stirring bar.
2. Add 3.7 mL of anhydrous DMF to the flask.
3. Slowly add 1.0 g of *tert*-butyldimethylchlorosilane (6.63 mmol) and 565 mg of imidazole (8.30 mmol) to above flask. Equip the round-bottom flask with an argon-filled balloon to protect the reaction against moisture. Stir the solution at room temperature (15°C–25°C) for 24 h.
4. Cool the reaction mixture to 0°C by using an ice-water cooling bath. Add 10 mL of distilled water to stop the reaction. Extract the mixture with ethyl acetate three times in a separatory funnel to afford the organic layer. Dry the organic layer with anhydrous magnesium sulfate (3 g) for 15 min and filter to remove the magnesium sulfate. Condense the organic layer using the rotary evaporator to afford the crude product. Characterize the product by ^1^H NMR spectroscopy (see the EXPECTED OUTCOMES section). Use the crude compound **1** to next reaction without further purification.

**Pause Point:** At this point, the product can be stored at -20°C for at least 3–6 months.

### Synthesis of 3-((*tert*-Butyldimethylsilyl)oxy)-*N,N*-diethyl-4-ethynylaniline (**2**)

**Timing: 24 h**

**Scheme 2:**
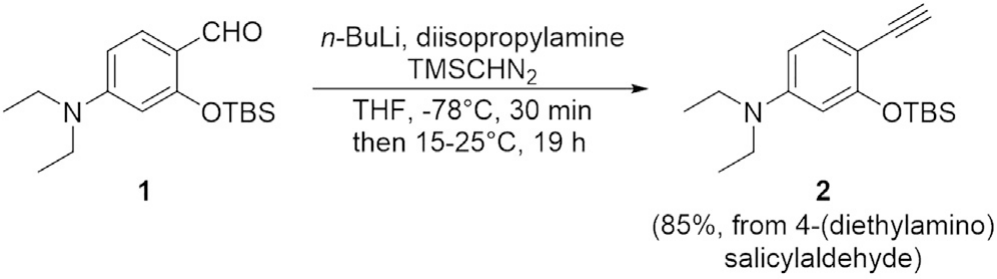
Synthesis of Compound **2**

This step describes how to prepare the compound **2**. See Scheme 2.

5. Slowly add a magnetic stirring bar to a 100 mL two-neck round-bottom flask. Equip the main neck of the two-neck round-bottom flask with an argon-filled balloon. Place a rubber septum on remaining side neck of the flask. This is the reaction flask.
6. Connect the flask to a vacuum pump. Put the flask under reduced pressure for 10 min and then back-fill the flask with argon.
7. Using a syringe, add 5.4 mL of anhydrous THF and 0.95 mL of diisopropylamine (6.77 mmol) through the septum on the side neck. Cool the flask to 0°C by using an ice-water cooling bath.
8. Using a syringe, add 3.1 mL of 2.0 M *n*-butyllithium in cyclohexane (6.25 mmol) dropwise to above flask. Stir the solution at 0°C for 10 min.
9. Cool the reaction mixture to -78°C by using a dry ice-acetone cooling bath. Using a syringe, add 3.1 mL of 2.0 M (trimethylsilyl)diazomethane in diethyl ether (6.25 mmol) dropwise to the flask. Stir the solution at -78°C for 30 min.
10. Charge a 25 mL two-neck round-bottom flask with a magnetic stirring bar and compound **1** (5.21 mmol). Equip the main neck of the two-neck round-bottom flask with an argon-filled balloon. Place a rubber septum on remaining side neck of the flask. Connect the flask to a vacuum pump. Put the flask under reduced pressure for 10 min and then back-fill the flask with argon.
11. Using a syringe, add 6.1 mL of anhydrous THF to the flask from Step 6. Stir the solution until the compound **1** has dissolved.
12. Using a syringe, add the compound **1** solution dropwise to the reaction flask from Step 5. Allow the reaction mixture to warm to room temperature (15°C–25°C) and stir the solution for 19 h.
13. Add 10 mL of distilled water to stop the reaction. Extract the mixture with chloroform three times in a separatory funnel to afford the organic layer. Dry the organic layer with anhydrous sodium sulfate (5 g) for 15 min and filter to remove the sodium sulfate. Condense the organic layer using the rotary evaporator to afford the crude product.
14. Purify it by NH_2_ silica gel column with hexane to obtain the pure compound **2** (1.35 g; yield = 85% from 4-(diethylamino)salicylaldehyde).
15. Characterize the product by NMR spectroscopy (^1^H NMR and ^13^C NMR) and electrospray ionization MS (HRMS, ESI) (see the EXPECTED OUTCOMES section). Troubleshooting 1

**CRITICAL:***n*-Butyllithium can burn in the presence of oxygen and moisture. Always use syringes with needles equipped with luer lock fittings when transferring *n*-butyllithium. Must avoid any spills with *n*-butyllithium and keep flammable solvent away to avoid a fire.

**CRITICAL:***n*-Butyllithium should be added slowly in a drop-by-drop manner.

**Pause Point:** At this point, the product can be stored at -20°C for at least 3–6 months.

### Synthesis of 3-(2-((*tert*-Butyldimethylsilyl)oxy)-4-(diethylamino)phenyl)propiolic Acid (**3**)

**Timing: 3 h**

**Scheme 3:**
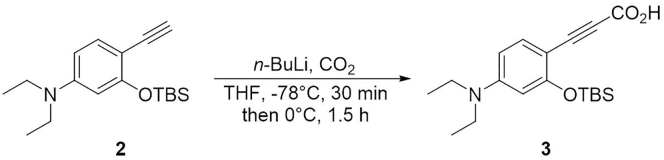
Synthesis of Compound **3**

This step describes how to prepare the compound **3**. See Scheme 3.

16. Weigh 243 mg of compound **2** (0.80 mmol) in a 50 mL two-neck round-bottom flask containing a magnetic stirring bar. Equip the main neck of the two-neck round-bottom flask with an argon-filled balloon. Place a rubber septum on remaining side neck of the flask. This is the reaction flask.
17. Connect the flask to a vacuum pump. Put the flask under reduced pressure for 10 min and then back-fill the flask with argon.
18. Using a syringe, add 3.2 mL of anhydrous THF through the septum on the side neck. Cool the flask to -78°C by using a dry ice-acetone cooling bath.
19. Using a syringe, add 0.57 mL of 2.0 M *n*-butyllithium in cyclohexane (0.88 mmol) dropwise to above flask. Stir the solution at -78°C for 30 min. Allow the reaction mixture to warm to 0°C by using an ice-water cooling bath.
20. Insert a cannula to the septum on the side neck and flush with CO_2_ for 10 min. Stir the solution at 0°C for 1.5 h.
21. Condense the organic layer using the rotary evaporator to afford the crude mixture. Characterize the product by electrospray ionization MS (HRMS, ESI) (see the EXPECTED OUTCOMES section). Use the crude compound **3** to next reaction without further purification. Troubleshooting 1 and 2

**CRITICAL:***n*-Butyllithium should be added slowly in a drop-by-drop manner. The condensation of organic layer should be run as quickly as possible, as the product is prone to degradation when it is left on water bath (35°C) for too long.

### Synthesis of 2-(2-(2-((3-(2-((*tert*-Butyldimethylsilyl)oxy)-4-(diethylamino)phenyl)propioloyl)oxy)ethoxy)ethoxy)ethyl (*R*)-4-((3*R*,5*R*,8*R*,9*S*,10*S*,13*R*,14*S*,17*R*)-3-Hydroxy-10,13-dimethylhexadecahydro-1*H*-cyclopenta[α]phenanthren-17-yl)pentanoate (**5**)

**Timing: 18 h**

**Scheme 4:**
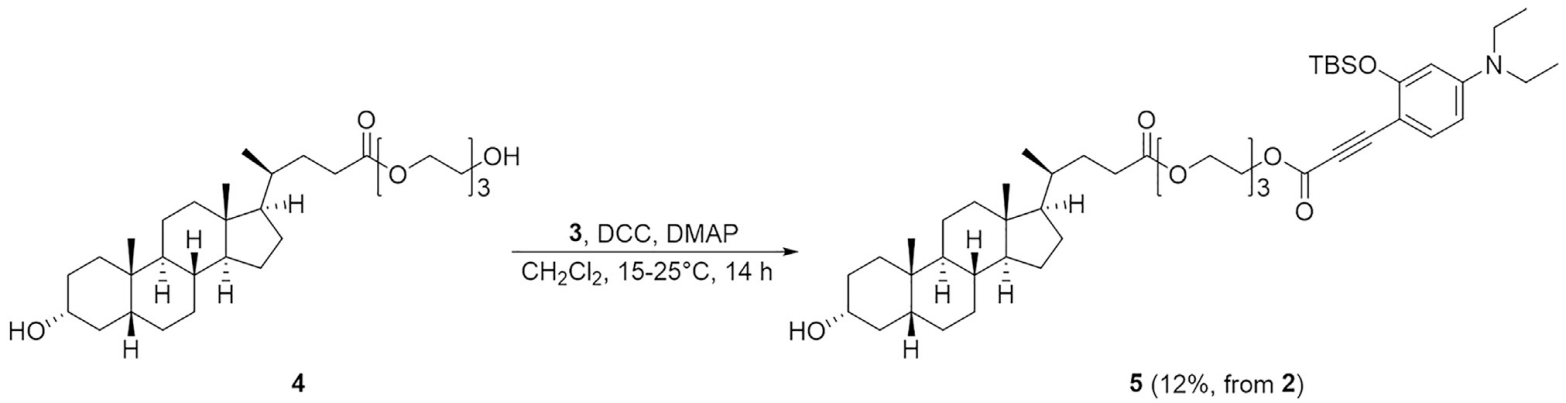
Synthesis of Compound **5**

This step describes how to prepare the compound **5**. See Scheme 4.

22. Weigh compound **3** (0.80 mmol) and 341 mg of lithocholic acid derivative (LCA) **4** (0.67 mmol) in a 25 mL two-neck round-bottom flask containing a magnetic stirring bar. Equip the main neck of the two-neck round-bottom flask with an argon-filled balloon. Place a rubber septum on remaining side neck of the flask. This is the reaction flask.
23. Connect the flask to a vacuum pump. Put the flask under reduced pressure for 10 min and then back-fill the flask with argon.
24. Using a syringe, add 0.8 mL of anhydrous CH_2_Cl_2_ through the septum on the side neck. Cool the flask to 0°C by using an ice-water cooling bath.
25. Charge a 10 mL two-neck round-bottom flask with a magnetic stirring bar and 177 mg of *N,N*′-dicyclohexylcarbodiimide (0.86 mmol). Equip the main neck of the two-neck round-bottom flask with an argon-filled balloon. Place a rubber septum on remaining side neck of the flask. Connect the flask to a vacuum pump. Put the flask under reduced pressure for 10 min and then back-fill the flask with argon.
26. Using a syringe, add 0.8 mL of anhydrous CH_2_Cl_2_ to the flask from Step 4.
27. Using a syringe, add the *N,N*′-dicyclohexylcarbodiimide solution and 0.74 mL of HCl solution (0.74 mmol, 1 M in ethyl acetate) dropwise to the reaction flask from Step 3. Add 9.4 mg of 4-dimethylaminopyridine (0.077 mmol) to the flask. Allow the reaction mixture to warm to room temperature (15°C–25°C) and stir the solution for 14 h.
28. Purify it by silica gel (sphere:40∼50 μm) column with 2:3 ethyl acetate/hexane to obtain the pure compound **5** (70 mg; yield = 12% from compound **2**).
29. Characterize the product by NMR spectroscopy (^1^H NMR and ^13^C NMR) and electrospray ionization MS (HRMS, ESI) (see the EXPECTED OUTCOMES section).

**Pause Point:** At this point, the product can be stored at -20°C for at least a week, but it should generally be used in the next step as soon as possible.

### Synthesis of 2-(2-(2-((3-(4-(Diethylamino)-2-hydroxyphenyl)propioloyl)oxy)ethoxy)ethoxy)ethyl (*R*)-4-((3*R*,5*R*,8*R*,9*S*,10*S*,13*R*,14*S*,17*R*)-3-Hydroxy-10,13-dimethylhexadecahydro-1*H*-cyclopenta[α]phenanthren-17-yl)pentanoate (LCA-TCC Probe **6**)

**Timing: 2 h**

**Scheme 5:**
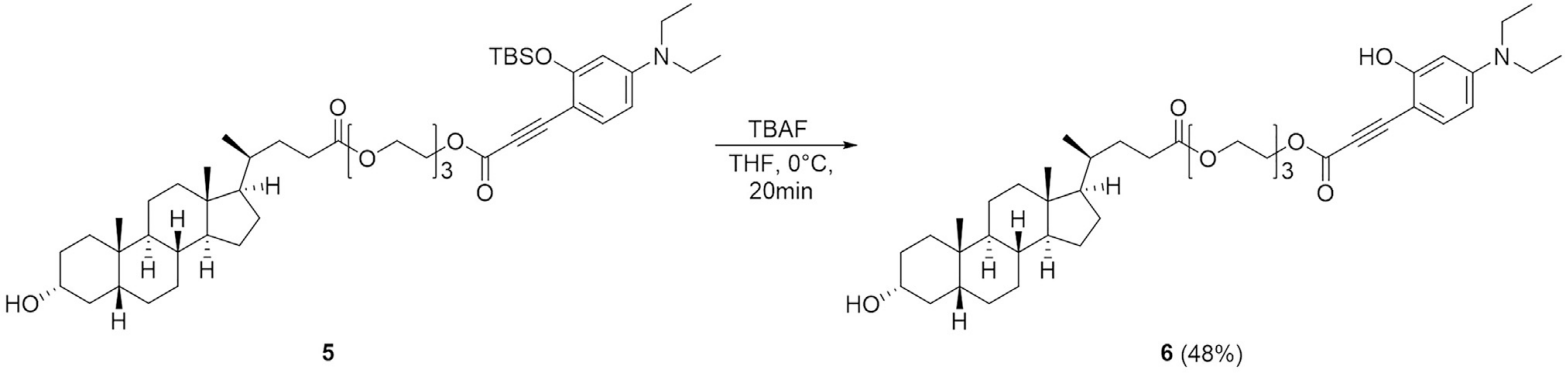
Synthesis of Lithocholic Acid-TCC Probe **6**

This step describes how to prepare the compound **6**. See Scheme 5.

30. Weigh 85 mg of compound **5** (0.10 mmol) in a 25 mL two-neck round-bottom flask containing a magnetic stirring bar. Equip the main neck of the two-neck round-bottom flask with an argon-filled balloon. Place a rubber septum on remaining side neck of the flask. This is the reaction flask.
31. Connect the flask to a vacuum pump. Put the flask under reduced pressure for 10 min and then back-fill the flask with argon.
32. Using a syringe, add 1.0 mL of anhydrous THF through the septum on the side neck. Cool the flask to 0°C by using an ice-water cooling bath.
33. Using a syringe, add 0.2 mL of tetrabutylammonium fluoride (0.2 mmol) dropwise to above flask. Stir the solution at 0°C for 20 min.
34. Purify it by silica gel (sphere:40∼50 μm) column with 3:2 ethyl acetate/hexane to obtain the pure compound **6** (35 mg; yield = 48%).
35. Characterize the product by NMR spectroscopy (^1^H NMR and ^13^C NMR) and electrospray ionization MS (HRMS, ESI) (see the EXPECTED OUTCOMES section). Troubleshooting 3

**CRITICAL:** The reaction reach completion in 20 min. To avoid decomposition of the product, do not leave the reaction mixture for longer than is necessary.

### Live-Cell Fluorescence Imaging of Exogenous Vitamin D Receptor in HeLa Cells Using LCA-TCC Probe **6**

**Timing: 72 h**

**Figure 1:**
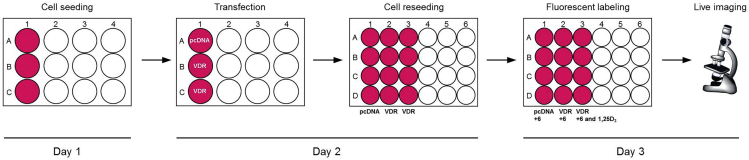
Workflow for Fluorescent Labeling of Vitamin D Receptor in HeLa Cells using TCC Probe **6**

This step describes how to fluorescently label target protein in living cells using TCC probe. See Figure 1.

36. Culture HeLa cells in a 10-cm cell culture dish containing 10 mL of culture medium in an incubator with 5% CO_2_ at 37°C.
37. Aspirate the culture medium from the cell culture dish using a vacuum pump and rinse the cells once with 5 mL of PBS.
38. Add 0.75 mL of trypsin-EDTA solution to the cells and place the cells into the 37°C incubator for 3 min.
39. Resuspend the cells in 10 mL of culture medium and count the number of cells under a microscope using a hemocytometer.
40. Seed 1 mL of the cell solution (containing 1 × 10^5^ cells) in a 12-well cell culture plate and place the plate in an incubator with 5% CO_2_ at 37°C for 8 h.
41. 70%–80% confluent is optimal for transfection with TransIT-LT1. For transient transfection of cells in a single well of a 12-well plate, add 100 μL of Opti-MEM, 1 μg of VDR-OFP plasmid and 3 μL of TransIT-LT1 to 1.5 mL microcentrifuge tube. As a negative control, add 100 μL of Opti-MEM, 1 μg of pcDNA3.1 plasmid and 3 μL of TransIT-LT1 to 1.5 mL microcentrifuge tube. Each solution should be mixed gently and incubated at room temperature (15°C–25°C) for 20 min.
42. The resultant lipoplex should be added dropwise to the cells and place the plate in an incubator with 5% CO_2_ at 37°C for 12 h.
43. Aspirate the culture medium from the cell culture dish using a vacuum pump and rinse the cells once with 1 mL of PBS.
44. Add 0.1 mL of trypsin-EDTA solution to the cells and place the cells into the 37°C incubator for 3 min.
45. Resuspend the cells in 1 mL of culture medium. Add 0.3 mL of cell culture medium and 0.2 mL of the cell solution on a poly-*L*-lysine coated cover slips in a 24-well cell culture plate. Place the plate in an incubator with 5% CO_2_ at 37°C for 24 h.
46. VDR-OFP expressing cells should be treated with LCA-TCC probe **6** at a concentration of 20 μM in 0.5 mL of cell culture medium. For competitive experiment, VDR-OFP expressing cells should be treated with LCA-TCC probe **6** and 1α,25-dihydroxyvitamin D_3_ at a concentration of 20 μM in 0.5 mL of cell culture medium. For negative control experiment, VDR-OFP untransfected cells should be treated with LCA-TCC probe **6** at a concentration of 20 μM in 0.5 mL of cell culture medium. Place the cells into the 37°C incubator for 8 h.
47. Transfer the cover slips to a glass bottom dish filled with DMEM/Ham’s F-12 culture medium. Take images using fluorescence microscope. Troubleshooting 4

**CRITICAL:** Cell culture medium should be prewarmed to 37°C. We recommend using lower-passage HeLa cells (< 20 passages) for live cell imaging experiments.

### Live-Cell Fluorescence Imaging of Mitochondria in HeLa Cells Using Tetramethylrhodamine Methyl Ester (TMRM) and LCA-TCC Probe **6**

**Timing: 26 h**

**Figure 2:**
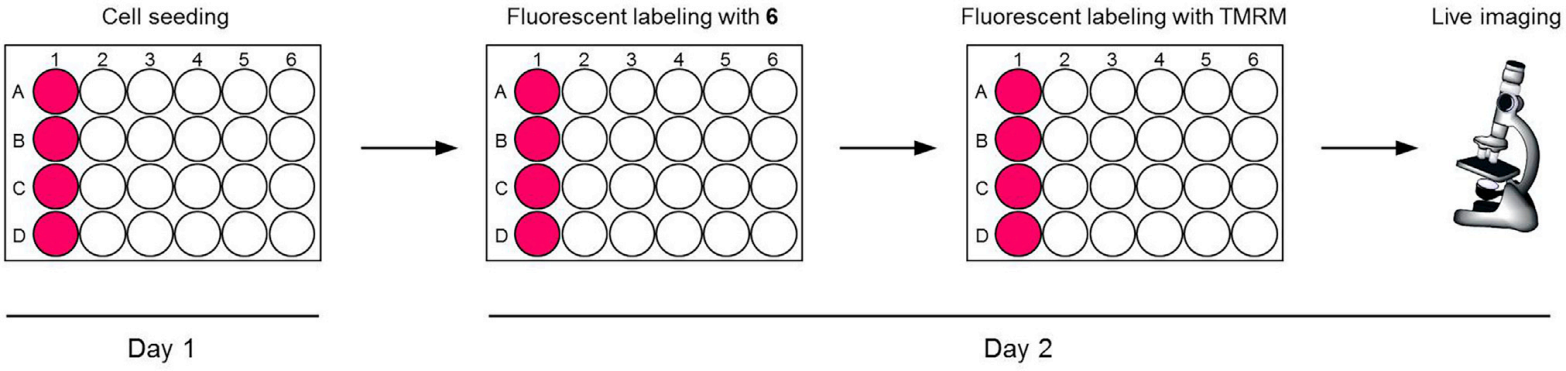
Workflow for Fluorescent Labeling of Mitochondria in HeLa Cells using Tetramethylrhodamine Methyl Ester (TMRM) and TCC Probe **6**

This step describes how to fluorescently label mitochondria in living cells. See Figure 2.

48. Culture HeLa cells in a 10-cm cell culture dish containing 10 mL of culture medium in an incubator with 5% CO_2_ at 37°C.
49. Aspirate the culture medium from the cell culture dish using a vacuum pump and rinse the cells once with 5 mL of PBS.
50. Add 0.75 mL of trypsin-EDTA solution to the cells and place the cells into the 37°C incubator for 3 min.
51. Resuspend the cells in 10 mL of culture medium and count the number of cells under a microscope using a hemocytometer.
52. Seed 0.5 mL of the cell solution (containing 5 × 10^4^ cells) on a poly-*L*-lysine coated cover slips in a 24-well cell culture plate. Place the plate in an incubator with 5% CO_2_ at 37°C for 24 h.
53. HeLa cells should be treated with LCA-TCC probe **6** at a concentration of 20 μM in 0.5 mL of cell culture medium. Place the cells into the 37°C incubator for 8 h.
54. Incubate labeled cells with 0.5 mL of cell culture medium containing 250 nM TMRM at 37°C for 30 min.
55. Rinse each cover slip three times with 0.5 mL of DMEM/Ham’s F-12 culture medium.
56. Transfer the cover slips to a glass bottom dish filled with DMEM/Ham’s F-12 culture medium. Take images using confocal microscopy.

**CRITICAL:** Cell culture medium should be prewarmed to 37°C.

## EXPECTED OUTCOMES

### Preparation of LCA-TCC Probe **6**

**Figure 3:**
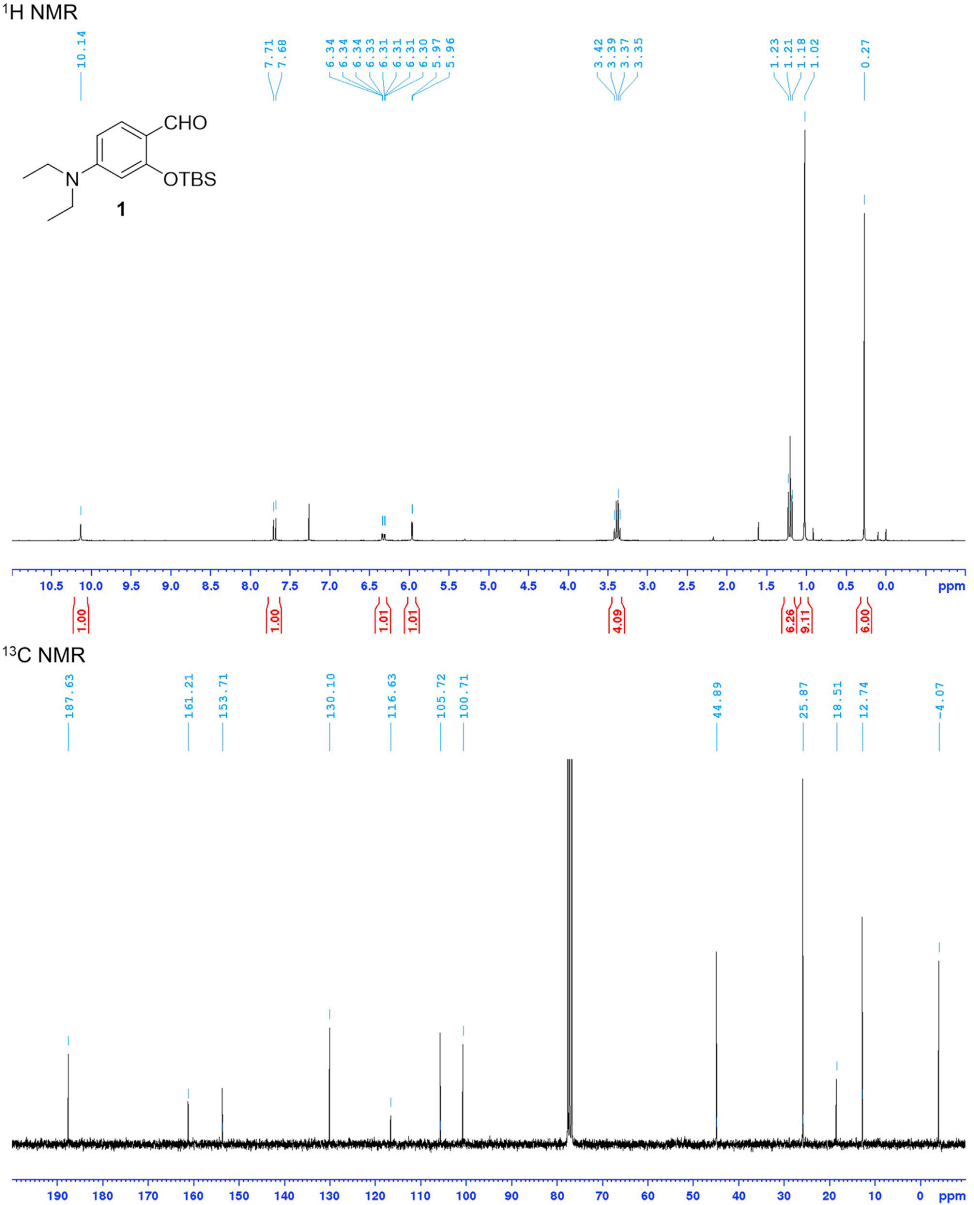
NMR Spectra of Compound **1**

**Figure 4:**
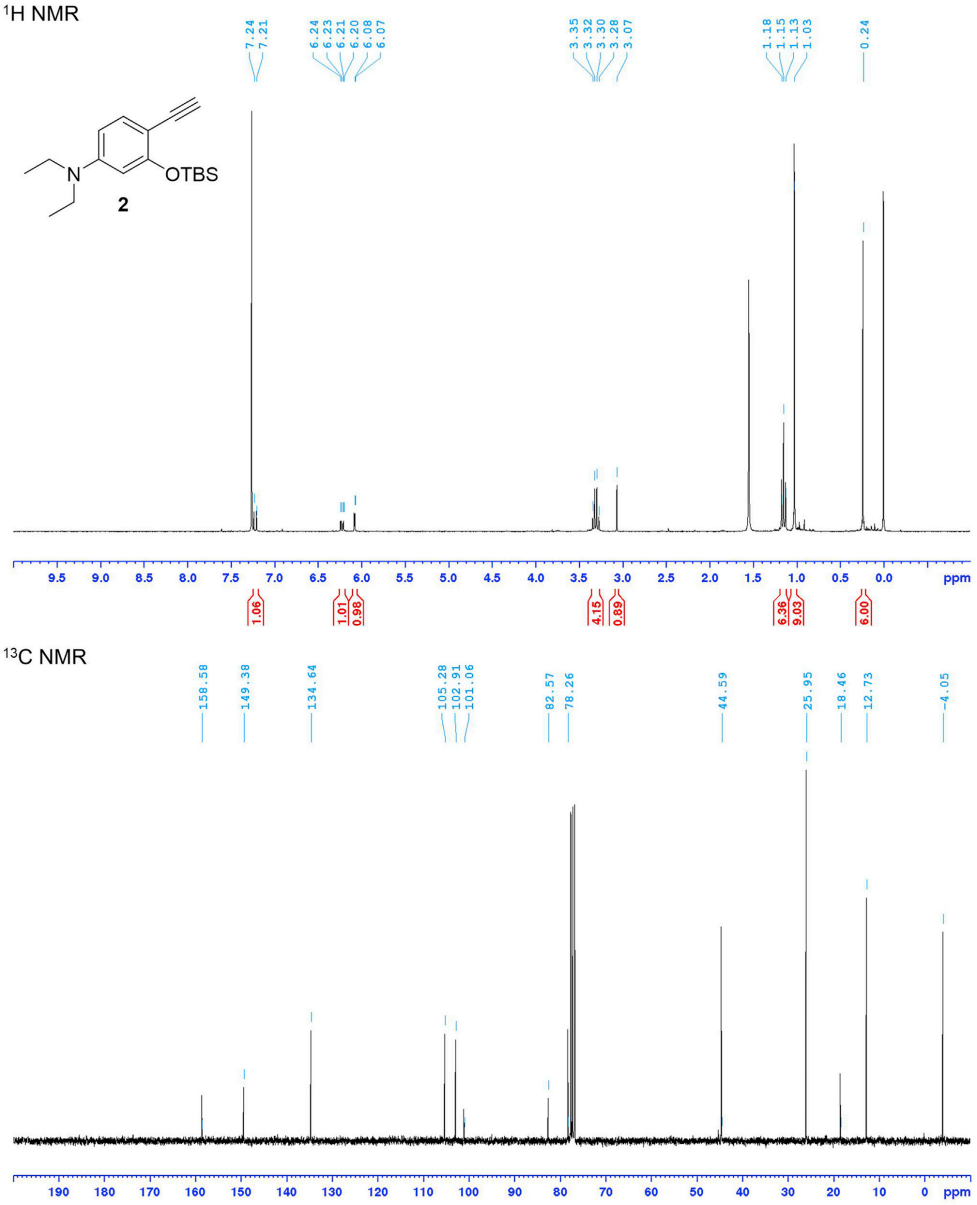
NMR Spectra of Compound **2**

**Figure 5:**
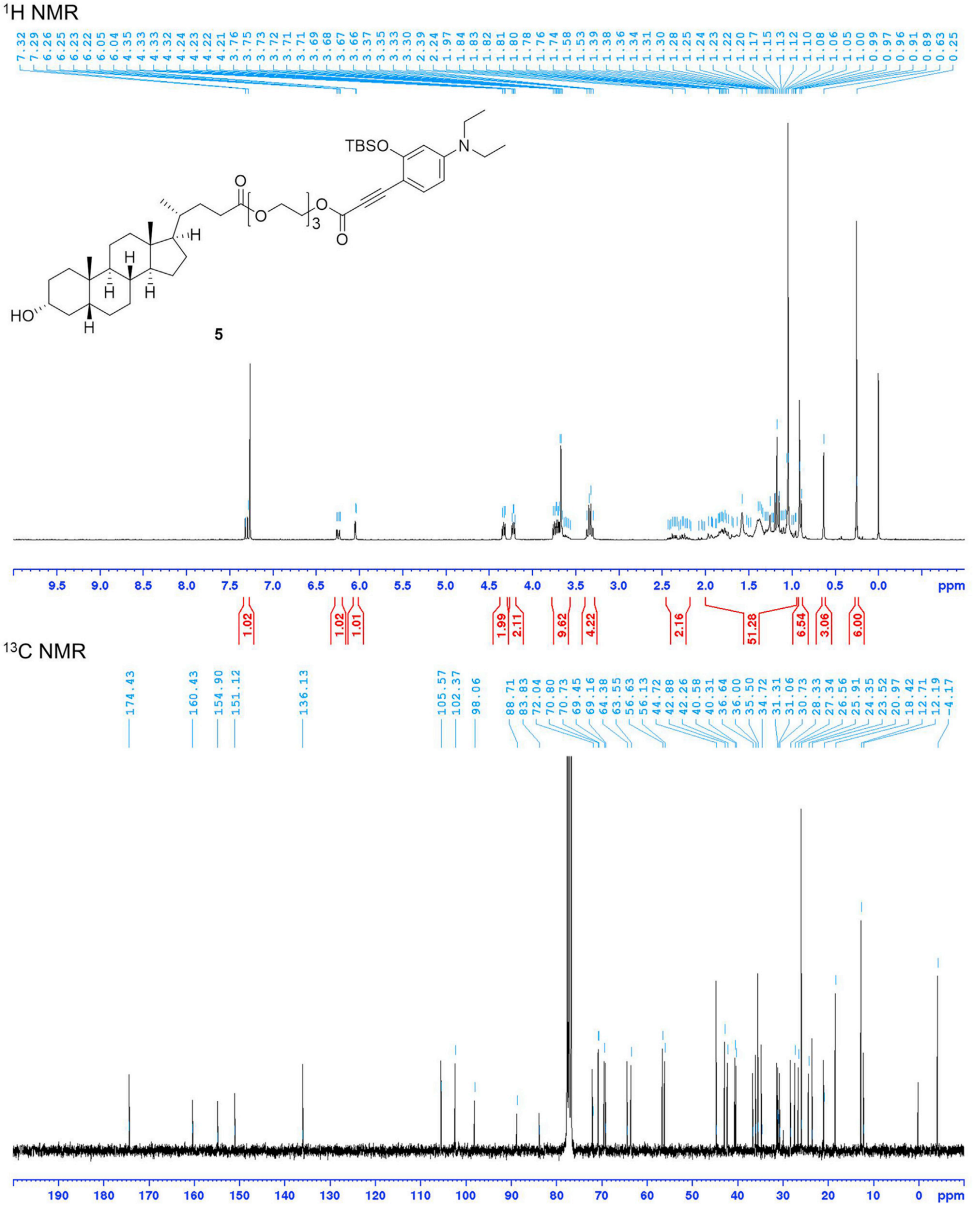
NMR Spectra of Compound **5**

**Figure 6:**
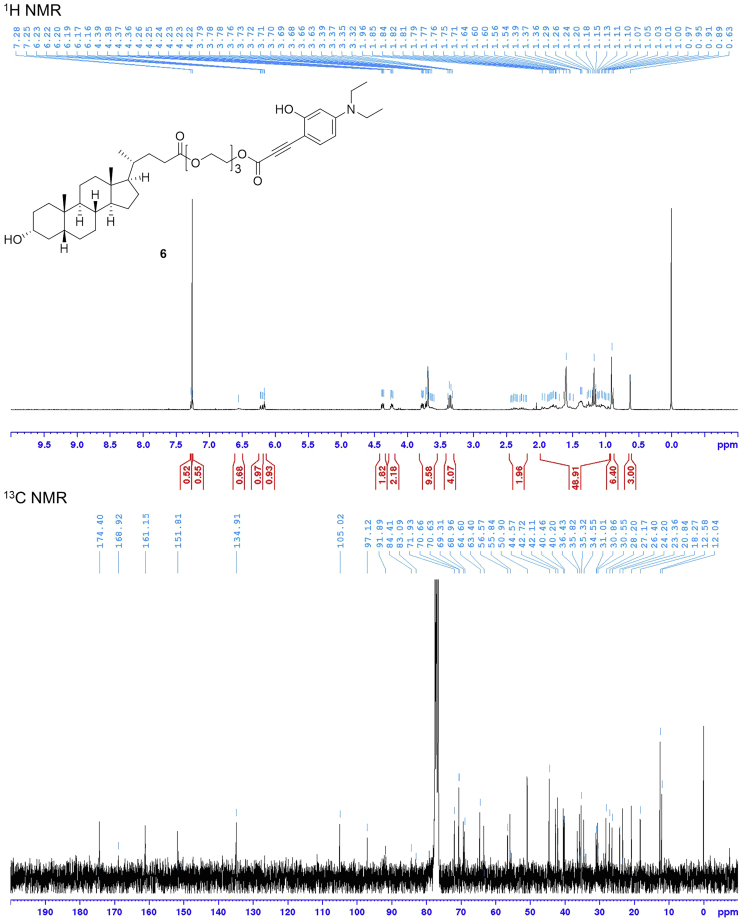
NMR Spectra of Compound **6**

Compound **6** is obtained by applying a five-step synthesis with an overall yield of 5%. The yields for compounds **1** and **3** were not determined because these compounds were used to next reaction without purification by silica gel column chromatography. Characterization data were summarized in Table 1. NMR spectra were shown in Figures 3, 4, 5, and 6.

**Table 1:** Analytical Data

Compound	Yield and R_f_ value	^1^H NMR (300 MHz, CDCl_3_) δ [ppm]	^13^C NMR (75 MHz, CDCl_3_) δ [ppm]	HRMS (ESI)
**1** (C_17_H_29_NO_2_Si)	R_f_ = 0.58 (ethyl acetate/hexane = 2:3)	10.14 (1H, s), 7.69 (1H, d, *J* = 9.0 Hz), 6.32 (1H, dd, *J* = 9.0, 2.4 Hz), 5.97 (1H, d, *J* = 2.4 Hz), 3.38 (4H, q, *J* = 7.1 Hz), 1.21 (6H, t, *J* = 7.1 Hz), 1.02 (9H, s), 0.27 (6H, s).	187.6, 161.2, 153.7, 130.1, 116.6, 105.7, 100.7, 44.9 (2 carbons), 25.9 (3 carbons), 18.5, 12.7 (2 carbons), -4.1 (2 carbons).	
**2** (C_18_H_29_NOSi)	85% from 4-(diethylamino)salicylaldehyde, R_f_ = 0.68 (ethyl acetate/hexane = 2:3)	7.22 (1H, d, *J* = 8.7 Hz), 6.22 (1H, dd, *J* = 8.7, 2.6 Hz), 6.08 (1H, d, *J* = 2.6 Hz), 3.31 (4H, q, *J* = 7.1 Hz), 3.07 (1H, s), 1.15 (6H, t, *J* = 7.1 Hz), 1.03 (9H, s), 0.24 (6H, s).	158.6, 149.4, 134.6, 105.3, 102.9, 101.1, 82.6, 78.3, 44.6 (2 carbons), 26.0 (3 carbons), 18.5, 12.7 (2 carbons), -4.1 (2 carbons).	Calcd. for C_18_H_30_NOSi [M + H]^+^ :304.20967, found :304.21007.
**3** (C_19_H_29_NO_3_Si)	R_f_ = 0.11 (ethyl acetate/hexane = 1:4)			Calcd. for C_19_H_30_NO_3_Si [M + H]^+^ :348.19949, found :348.20495.
**5** (C_49_H_79_NO_8_Si)	12% from compound **2**, R_f_ = 0.7 (ethyl acetate/hexane = 7:3)	7.31 (1H, d, *J* = 8.8 Hz), 6.24 (1H, dd, *J* = 8.8, 2.5 Hz), 6.04 (1H, d, *J* = 2.5 Hz), 4.35–4.32 (2H, m), 4.24–4.21 (2H, m), 3.76–3.57 (9H, m), 3.34 (4H, q, *J* = 7.1 Hz), 2.44–2.17 (2H, m), 1.97–0.96 (42H, overlapped), 0.91–0.89 (6H, overlapped), 0.63 (3H, s), 0.25 (6H, s).	174.4, 160.4, 154.9, 151.1, 136.1, 105.6, 102.4, 98.1, 88.7, 83.8, 72.0, 70.8, 70.7, 69.4, 69.2, 64.4, 63.6, 56.6, 56.1, 44.7 (2 carbons), 42.9, 42.3, 40.6, 40.3, 36.6, 36.0, 35.5 (2 carbons), 34.7, 31.3, 31.1, 30.7, 28.3, 27.3, 26.6, 25.9 (3 carbons), 24.4, 23.5, 21.0, 18.4 (2 carbons), 12.7 (2 carbons), 12.2, -4.2 (2 carbons).	Calcd. for C_49_H_80_NO_8_Si [M + H]^+^ :838.56532, found :838.56186.
**6** (C_43_H_65_NO_8_)	48%, R_f_ = 0.35 (ethyl acetate/hexane = 7:3)	7.27 (1H, d, *J* = 8.7 Hz), 6.56 (1H, brs), 6.21 (1H, dd, *J* = 8.7, 2.5 Hz), 6.17 (1H, d, *J* = 2.5 Hz), 4.39–4.36 (2H, m), 4.26–4.22 (2H, m), 3.79–3.60 (9H, m), 3.36 (4H, q, *J* = 7.1 Hz), 2.44–2.20 (2H, m), 1.96–0.95 (33H, overlapped), 0.91–0.89 (6H, overlapped), 0.63 (3H, s).	174.4, 168.9, 161.1, 151.8, 134.9, 105.0, 97.1, 91.9, 84.4, 83.1, 71.9, 70.7, 70.6, 69.3, 69.0, 64.6, 63.4, 56.6, 55.8, 50.9, 44.6 (2 carbons), 42.7, 42.1, 40.5, 40.2, 36.4, 35.8, 35.3, 34.5, 31.0, 30.9, 30.5, 28.2, 27.2, 26.4, 24.2, 23.4, 20.8, 18.3, 12.6 (2 carbons), 12.0.	Calcd. for C_43_H_66_NO_8_ [M + H]^+^ :724.47884, found :724.47512.

Analytical data for 2-((*tert*-Butyldimethylsilyl)oxy)-4-(diethylamino)benzaldehyde (**1**), 3-((*tert*-Butyldimethylsilyl)oxy)-*N,N*-diethyl-4-ethynylaniline (**2**), 3-(2-((*tert*-Butyldimethylsilyl)oxy)-4-(diethylamino)phenyl)propiolic acid (**3**), 2-(2-(2-((3-(2-((*tert*-Butyldimethylsilyl)oxy)-4-(diethylamino)phenyl)propioloyl)oxy)ethoxy)ethoxy)ethyl (*R*)-4-((3*R*,5*R*,8*R*,9*S*,10*S*,13*R*,14*S*,17*R*)-3-hydroxy-10,13-dimethylhexadecahydro-1*H*-cyclopenta[α]phenanthren-17-yl)pentanoate (**5**), and 2-(2-(2-((3-(4-(Diethylamino)-2-hydroxyphenyl)propioloyl)oxy)ethoxy)ethoxy)ethyl (*R*)-4-((3*R*,5*R*,8*R*,9*S*,10*S*,13*R*,14*S*,17*R*)-3-hydroxy-10,13-dimethylhexadecahydro-1*H*-cyclopenta[α]phenanthren-17-yl)pentanoate (**6**).

### Live-Cell Fluorescence Imaging of Exogenous Vitamin D Receptor in HeLa Cells using LCA-TCC Probe **6**

**Figure 7:**
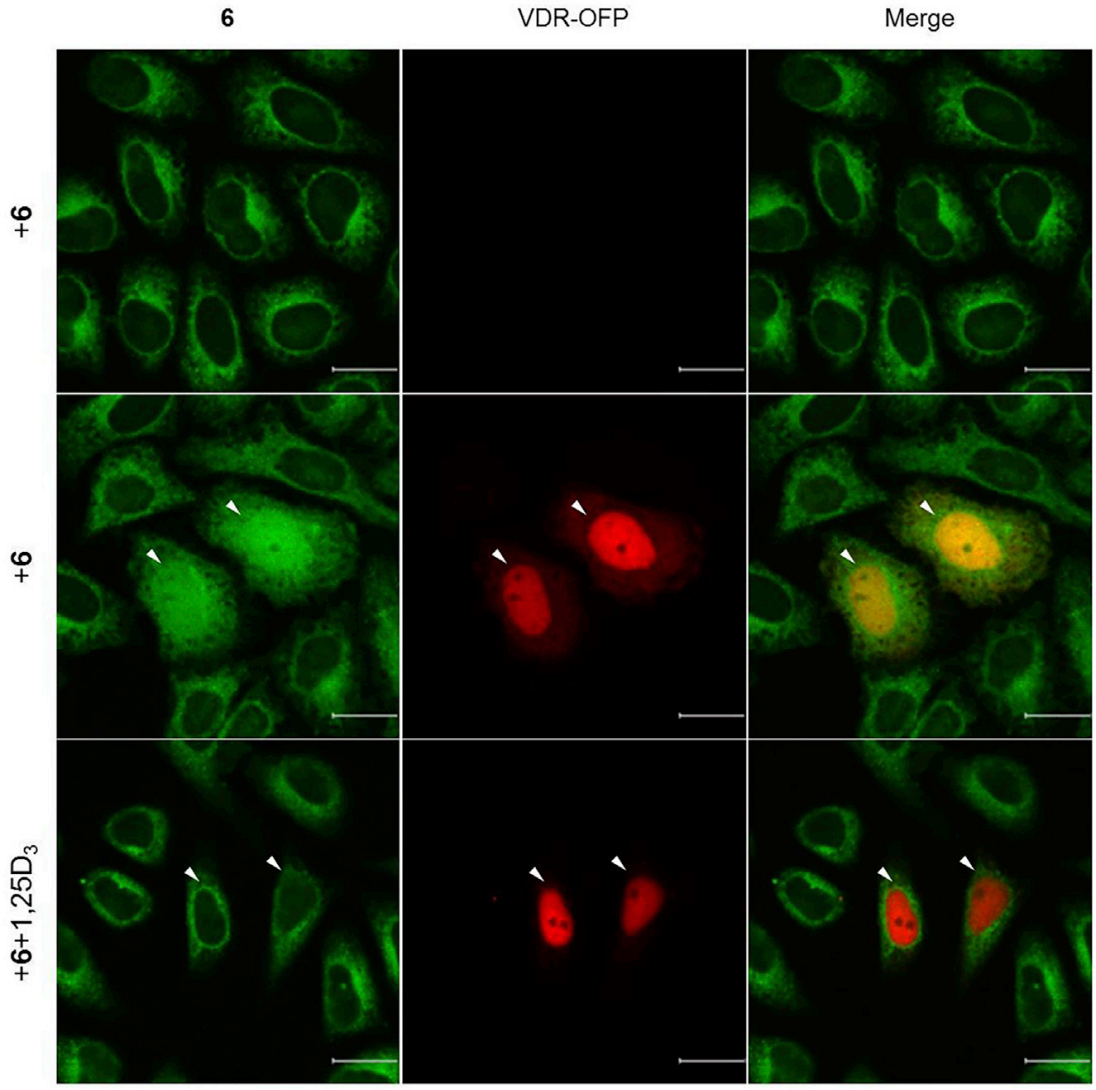
Live-cell Fluorescence Imaging of Exogenous Vitamin D Receptor in HeLa Cells Using LCA-TCC Probe **6** Upper panel: HeLa cells not expressing vitamin D receptor-orange fluorescent protein (VDR-OFP) were treated with **6**. Middle panel: HeLa cells expressing VDR-OFP were treated with **6**. Lower panel: HeLa cells expressing VDR-OFP were treated with **6** in the presence of 1,25D_3_. Arrowheads indicate VDR-OFP expressing HeLa cells. Scale bars, 20 μm.

**Figure 8:**
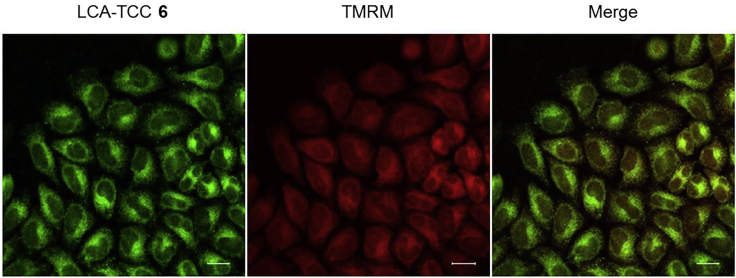
Live-cell Fluorescence Imaging of Mitochondria in HeLa Cells using TMRM and LCA-TCC Probe **6** Scale bars, 20 μm.

As shown in Figure 7, the addition of lithocholic acid (LCA)-TCC probe **6** results in weak fluorescence signals in the cytoplasm of HeLa cells not expressing vitamin D receptor-orange fluorescent protein (VDR-OFP). These signals colocalize with tetramethylrhodamine methyl ester (TMRM) signals of mitochondrial marker (Figure 8). In yeast Saccharomyces cerevisiae, LCA accumulates in mitochondria and extends the chronological lifespan (Beach et al., 2015). LCA alters mitochondrial function in PC-3 and DU-145 prostate cancer cells (Gafar et al., 2016). Unlike negative control experiment, nuclear fluorescence signals are observed in cells expressing VDR-OFP, and these fluorescence signals overlapped well with the VDR-OFP signals. The nuclear signals are completely diminished in the presence of 1α,25-dihydroxyvitamin D_3_ (1,25D_3_). We successfully used the LCA-TCC probe **6** for live-cell imaging of vitamin D receptor. The fluorogenic behavior of LCA-TCC probe is expected to enable direct live-cell imaging by simple incubation of the LCA-TCC probe with HeLa cells in a single step and without the need for any washing operation.

## LIMITATIONS

Structure of ligand unit will be limited to avoid intramolecular reaction of fluorophore unit with highly nucleophilic functional groups. To avoid undesired reaction, we recommend not to use a ligand having extra reactive thiol or amino group.

LCA-TCC probe **6** fluorescently labels not only vitamin D receptor but also mitochondria, which is unfavorable for accurate evaluation of target protein’s distribution. Although LCA is a low-affinity ligand for vitamin D receptor (inhibition constant (Ki) = 29 ± 6 μM in COS-7 cells) (Makishima et al., 2002), LCA-TCC probe **6** can label vitamin D receptor in living cells. The target selectivity of TCC probe will be improved to use high-affinity and selective ligand unit. Fluorescent labeling selectivity can be ligand unit dependent.

## TROUBLESHOOTING

### Problem 1

Insufficient yield of compound **2** and **3**.

### Potential Solution

*n*-Butyllithium is moisture sensitive and is gradually decomposed by reacting with moisture. Estimate the concentration of *n*-butyllithium by using diphenylacetic acid titration (Kofron and Baclawski, 1976) before it is used.

### Problem 2

Insufficient yield of compound **3**.

### Potential Solution

Compound **3** is unstable for heat and light. Cover flask with aluminum sheet during the reaction and evaporation. Condense the organic solvent using the rotary evaporator immediately and use the crude product for the next reaction on the day.

### Problem 3

Insufficient yield of LCA-TCC probe **6**.

### Potential Solution

LCA-TCC probe **6** is degraded during the deprotection reaction for *tert*-butyldimethylsilyl ether protecting group. Reduce reaction time or reaction temperature.

### Problem 4

Highly nonspecific staining.

### Potential Solution

Reduce LCA-TCC probe **6** concentration and wash out excess probe with PBS. As an alternative approach, optimize ligand unit selectivity and affinity for protein of interest.

### Problem 5

To design TCC probe, there are no information about interaction between ligand and target protein.

### Potential Solution

We recommend adding fluorophore unit to polar side of the ligand. Because polar side of ligand often faces to surface of protein where many types of nucleophilic amino acid residues exist.

## References

[bib1] Beach A., Richard V.R., Bourque S., Boukh-Viner T., Kyryakov P., Gomez-Perez A., Arlia-ciommo A., Feldman R., Leonov A., Piano A. (2015). Lithocholic bile acid accumulated in yeast mitochondria orchestrates a development of an anti-aging cellular pattern by causing age-related changes in cellular proteome. Cell Cycle.

[bib2] Gafar A.A., Draz H.M., Goldberg A.A., Bashandy M.A., Bakry S., Khalifa M.A., Abushair W., Titorenko V.I., Sanderson J.T. (2016). Lithocholic acid induces endoplasmic reticulum stress, autophagy and mitochondrial dysfunction in human prostate cancer cells. PeerJ.

[bib3] Kofron W.G., Baclawski L.M. (1976). A convenient method for estimation of alkyllithium concentrations. J. Org. Chem..

[bib4] Kojima H., Fujita Y., Takeuchi R., Ikebe Y., Ohashi N., Yamamoto K., Itoh T. (2020). Cyclization reaction-based turn-on probe for covalent labeling of target proteins. Cell Chem. Biol..

[bib5] Makishima M., Lu T.T., Xie W., Whitfield G.K., Domoto H., Evans R.M., Haussler M.R., Mangelsdorf D.J. (2002). Vitamin D receptor as an intestinal bile acid sensor. Science.

[bib6] Wang C., Yang S., Yi M., Liu C., Wang Y., Li J., Li Y., Yang R. (2014). Graphene oxide assisted fluorescent chemodosimeter for high-performance sensing and bioimaging of fluoride lons. Appl. Mater. Interfaces.

